# Effects of two exercise modalities and subsequent detraining on trabecular bone microarchitecture in growing male rats

**DOI:** 10.14814/phy2.70941

**Published:** 2026-05-27

**Authors:** Yong‐In Ju, Teruki Sone

**Affiliations:** ^1^ Department of Health and Sports Sciences Kawasaki University of Medical Welfare Kurashiki Okayama Japan; ^2^ Department of Radiological Technology Kawasaki University of Medical Welfare Kurashiki Okayama Japan

**Keywords:** bone health, detraining, jump exercise, micro–computed tomography, trabecular microarchitecture, treadmill running

## Abstract

Weight‐bearing and high‐impact exercise during growth enhance bone accrual; however, the extent to which these benefits persist after detraining remains unclear. This study examined the effects of detraining after treadmill running and jump exercise on trabecular bone microarchitecture and strength in growing rats. Ninety male Wistar rats were assigned to control, running, or jump groups (*n* = 30 each) and trained for 8 weeks, followed by 0, 12, or 24 weeks of detraining. Running was performed at 25 m/min for 60 min/day, and jump training consisted of 10 jumps/day (40 cm), both conducted 5 days/week. Distal femoral trabecular microarchitecture, volumetric bone mineral density (vBMD), and finite element analysis–derived fracture load were assessed using micro‐computed tomography. Both exercise modalities increased trabecular bone volume fraction, thickness, number, vBMD, and fracture load compared with controls (all *p* < 0.05–0.001). Jump exercise preferentially increased trabecular thickness, whereas running was associated with higher trabecular number. These benefits were largely maintained for up to 12 weeks after exercise cessation but were no longer evident after 24 weeks of detraining. These findings suggest that exercise‐induced improvements in trabecular bone during growth are partially lost following exercise cessation, suggesting continued mechanical loading is required to maintain these adaptations.

## Graphical abstract

First to delineate detraining effects on trabecular microarchitecture in growing rats. Running was associated with higher trabecular number, whereas jumping chiefly increased thickness. Gains in bone mass and strength persisted for up to 12 weeks post‐exercise but regressed to control by 24 weeks. Continuous physical activity appears necessary to preserve skeletal adaptations and support long‐term bone health.

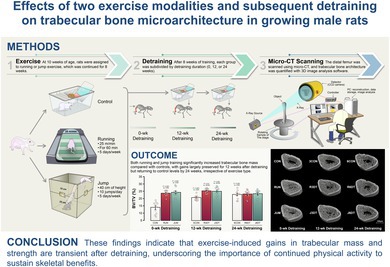

## 1 INTRODUCTION

Weight‐bearing, high‐impact exercise is widely recommended for osteoporosis prevention because it enhances bone mass and strength during periods of rapid growth (Nikander et al., 2010). Achieving higher peak bone mass during growth is considered a major determinant of osteoporosis prevention and reduced fracture risk later in life, particularly when skeletal gains are maintained after exercise cessation (Zhu & Zheng, 2021).

The effects of detraining on exercise‐induced bone adaptations remain controversial. Numerous studies in humans (Dalsky et al., 1988; Gustavsson et al., 2003; Iwamoto et al., 2001; Kudlac et al., 2004; Nilsson et al., 2014; Nordström et al., 2005; Valdimarsson et al., 2005; Vuori et al., 1994; Winters & Snow, 2000) and animal models (Honda et al., 2008; Iwamoto et al., 2000; LeBlanc et al., 1983; Ooi et al., 2011; Pajamäki et al., 2003; Umemura et al., 2008; Warden et al., 2007; Wu et al., 2004; Yeh & Aloia, 1990) have reported varying degrees of bone mass loss following exercise cessation. For example, several human studies have shown that bone mass gained through physical activity can be largely lost within 3–13 months of detraining (Dalsky et al., 1988; Iwamoto et al., 2001; Vuori et al., 1994; Winters & Snow, 2000). Similar findings have been reported in animal studies (Iwamoto et al., 2000; LeBlanc et al., 1983; Ooi et al., 2011; Pajamäki et al., 2003; Wu et al., 2004; Yeh & Aloia, 1990); in rats, for instance, bone mass acquired from treadmill running was lost after just 4 weeks of exercise cessation (Iwamoto et al., 2000; Yeh & Aloia, 1990). In contrast, other studies have shown that bone mass and strength gained from jump exercise are relatively well maintained after detraining. In rats, 8 weeks of jump exercise produced gains that persisted for up to 24 weeks, despite some decline (Honda et al., 2008; Umemura et al., 2008). Similar observations have been reported in human trials, where bone mass achieved through high‐impact activities remained largely stable over comparable detraining periods (Dalsky et al., 1988; Nilsson et al., 2014; Nordström et al., 2005). These differences in findings could reflect variations in exercise type, skeletal site, and assessment methods. Accordingly, it is plausible that the rate of bone mass loss following exercise cessation differs between running and jump exercises.

In line with these findings, previous studies have reported that bone mass and strength gained from jump training were preserved after detraining in young, adult, and ovariectomized rats, and these effects were accompanied by morphometric changes in bone (Honda et al., 2008; Umemura et al., 2008). These morphometric changes, such as alterations in trabecular thickness and connectivity, indicate that the microarchitecture of trabecular bone may influence how rapidly bone mass is lost following exercise cessation. Consequently, even when overall bone mass is comparable, differences in trabecular microstructure may contribute to the observed variation in the rate of bone mass loss. We previously demonstrated that both running and jump exercises during the remobilization period following suspension‐induced osteopenia could restore femoral trabecular architecture in young growing rats (Ju et al., 2012). Interestingly, the effects on cancellous bone differed between the two exercise modalities: jump exercise primarily increased trabecular bone volume by thickening existing trabeculae, whereas treadmill running predominantly augmented bone by forming new trabeculae. This observation aligns with previous findings in mice, where treadmill running and targeted tibial loading exerted distinct effects on trabecular bone structure (Berman et al., 2019). Thus, different forms of mechanical loading imposed by running and jump exercise may lead to unique patterns of bone adaptation. Furthermore, variations in trabecular bone surface area may influence the maintenance of bone mass after exercise cessation. Taken together, these findings support a direct comparison of skeletal responses to exercise cessation between running and jump exercises, which has not yet been examined.

Accordingly, this study aimed to compare exercise‐induced trabecular bone microarchitectural adaptations between running and jump exercise, and to examine how these adaptations respond to detraining. We hypothesized that jump exercise would primarily induce trabecular thickening, whereas running would predominantly increase trabecular number, and that the thickening response induced by jump exercise would be more resistant to detraining, leading to greater long‐term preservation of trabecular bone mass compared with running.

## 2 MATERIALS AND METHODS

### 2.1 Animals and experimental design

**FIGURE 1:**
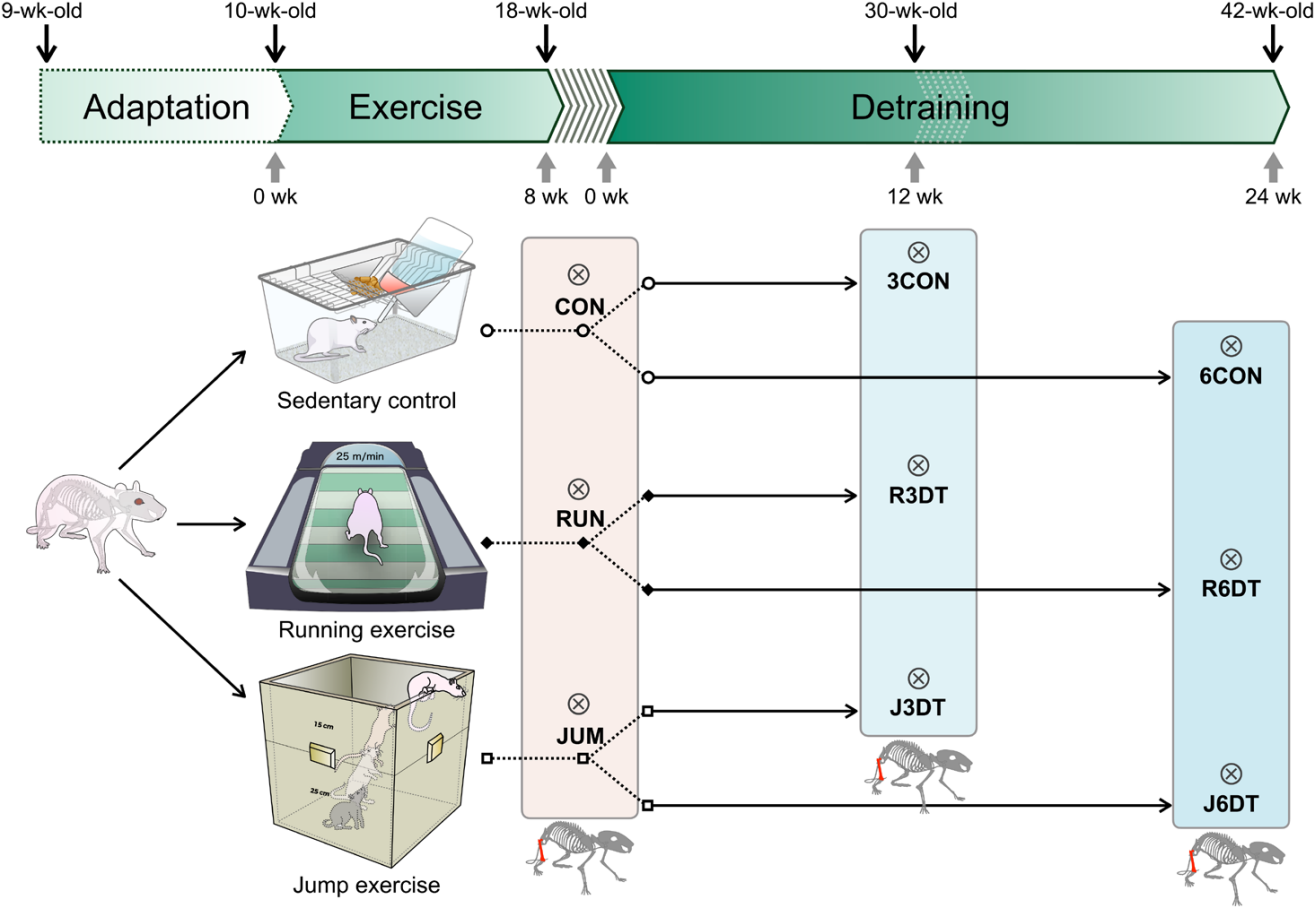
Experimental flowchart showing the exercise and detraining schedule. Rats began running or jump exercise at 10 weeks of age and continued for 8 weeks. Rats were sacrificed either immediately after training (18 weeks old) or after 12 or 24 weeks of detraining, corresponding to final ages of 30 and 42 weeks, respectively. Abbreviations indicate experimental groups used throughout all figures and tables: CON, 8‐week sedentary control; RUN, 8 weeks of running exercise (5 days/week, 25 m/min, 1 h/day); JUM, 8 weeks of jump exercise (5 days/week, 10 jumps/day, 40 cm height); 3CON, 30‐week sedentary control; R3DT and J3DT, exercise followed by 12 weeks of detraining; 6CON, 42‐week sedentary control; R6DT and J6DT, exercise followed by 24 weeks of detraining. These abbreviations are applied consistently across all figures and tables in this study. Symbols indicate experimental conditions as follows: ⊗, dissection; DT, detraining; ○, sedentary control; ♦, running exercise; □, jump exercise.

Ninety male Wistar rats (9 weeks old) were obtained from CLEA Japan (Osaka, Japan) and acclimated for 1 week. All rats were individually housed in plastic cages (20 × 33 × 14 cm) in a temperature‐controlled room (22°C ± 1°C) under a 12:12 h light/dark cycle. The rats were fed a standard commercial diet (MF; Oriental Yeast Co., Ltd., Chiba, Japan) containing 1.15% calcium and 0.88% phosphorus. Body weight and food intake were recorded daily before each exercise session. Water was provided ad libitum. All experimental procedures and animal care were conducted in accordance with the guidelines of the Kawasaki University of Medical Welfare Laboratory Animal Care and Use Committee (Permit No. 10‐011). Figure 1 illustrates a detailed experimental procedure of the study including the training and detraining periods, grouping, and age of rats. Briefly, rats were habituated to the diet and new environment for 1 week. First, rats were randomly assigned to three groups. Group 1 served as the sedentary control group (*n* = 30), Group 2 performed running exercise (*n* = 30), and Group 3 performed jump exercise for 8 weeks (*n* = 30). After completion of the 8‐week exercise period, three groups were further divided into three subgroups according to the duration of exercise cessation, resulting in a total of nine groups: RUN, running exercise for 8 weeks; JUM, jump exercise for 8 weeks; CON, sedentary control for 8 weeks; R3DT, running exercise followed by 12 weeks of detraining; J3DT, jump exercise followed by 12 weeks of detraining; 3CON, sedentary control for 12 weeks; R6DT, running exercise followed by 24 weeks of detraining; J6DT, jump exercise followed by 24 weeks of detraining; and 6CON, sedentary control for 24 weeks. Rats were euthanized under isoflurane anesthesia after the 8‐week exercise period and at the end of each detraining phase. All efforts were made to minimize animal suffering. Immediately after euthanasia, the right calf muscles were collected from each rat and weighed. The excised femora were cleared of soft tissue, and femoral length was measured using digital calipers. The right femora were then stored at −40°C until analysis by micro‐computed tomography (micro‐CT) (Ju et al., 2008, 2012, 2014, 2020, 2022).

### 2.2 Exercise conditions

Rats in the running exercise group were trained on a motorized treadmill (KN‐73 Tread‐Mill RM‐5; Natume, Tokyo, Japan). Initially, the rats ran for 10 min per day for 5 consecutive days at a low speed of 10 m/min with a 0° incline. As the rats adapted to the treadmill, both speed and duration were gradually increased until the animals could run at 25 m/min for 60 min/day, which was then maintained for the remainder of the training program. This graduated protocol is suitable as a chronic aerobic exercise model in rats, as previously reported (Joo et al., 2003). No electrical stimulus was applied during running; instead, compressed air was blown from behind to encourage movement. To enhance activity, the front half of the treadmill was covered with black paper to create a darkened environment.

The jump exercise program was conducted according to our previous publications (Ju et al., 2008, 2012, 2014, 2020, 2022) and consisted of 10 jumps per day, 5 days per week for 8 weeks. Briefly, each rat in the jumping exercise group was placed individually at the bottom of a specially designed wooden box (40 × 40 × 40 cm). Initially, an electrical stimulus was applied to encourage the rat to jump, grasp the top of the box with its forelimbs, and climb up. The rat was then immediately returned to the floor to repeat the procedure, ensuring that the ground reaction force at landing did not exceed body weight. As the rats became accustomed to the training, the electrical stimulus was no longer necessary. The height of the box was adjustable, initially set at 25 cm and progressively increased to 40 cm during the first week. Completing 10 jumps required approximately 1 min.

### 2.3 Micro‐CT imaging and 3D architectural indices

Femurs were scanned using micro‐CT (Ele Scan mini; Nittetsu Elex, Tokyo, Japan) at 30 kVp and 80 μA with a 0.1‐mm copper filter. Images were reconstructed into three‐dimensional datasets with an isotropic voxel size of 19.3 μm (512 × 512 matrix) for trabecular bone morphometric analyses, as previously described (Joo et al., 2003; Ju et al., 2008, 2012, 2014, 2020, 2022). Trabecular bone was scanned 2.8–3.0 mm proximal to the distal femoral end, including the metaphysis–growth plate border. A total of 300 slices (19.3 μm, ~5.5 mm) were acquired, with the volume of interest defined as the 130 slices above the proximal growth plate. BMD values were calibrated using a hydroxyapatite phantom (6 × 1 mm; 200–800 mg/cm^3^; Kyoto Kagaku, Kyoto, Japan), which was scanned under the same conditions as the bone samples. Trabecular bone volume fraction (BV/TV, %), trabecular thickness (Tb.Th, μm), trabecular number (Tb.N, 1/mm), trabecular separation (Tb.Sp, μm), connectivity density (Conn.D, 1/mm^3^), trabecular bone pattern factor (TBPf, 1/mm), structure model index (SMI), and volumetric bone mineral density (vBMD, mg/cm^3^) were calculated using TRI/3D‐Bon BMD software (Ratoc System Engineering, Tokyo, Japan).

### 2.4 Micro‐finite element analysis (micro‐FEA) for micro‐CT images

Micro‐FEA was performed using TRI/3D‐FEM64 software (RATOC System Engineering, Tokyo, Japan) on distal femoral reconstructions from micro‐CT (130 slices, voxel size: 18.11 μm), as previously described (Ju et al., 2020, 2022). Compression was simulated by fixing the proximal surface and applying a 400 N load perpendicular to the distal surface. Trabecular bone was modeled as isotropic and linearly elastic (Poisson's ratio = 0.3), with Young's modulus (E, MPa) derived from Carter's equation (Carter & Hayes, 1977): *E* = 16.311 × 10^−5^ × [mineralization density]^3^. Fracture load was defined as the point at which 2.8% of elements exceeded a shear stress of 68 MPa, and the total reaction force on the fixed proximal surface was recorded.

### 2.5 Sample size determination

The minimum number of animals per group was set at 10 based on previous data from our laboratory and related studies. Sample size adequacy was further evaluated using G*Power software (version 3.1; Heinrich Heine University Düsseldorf, Germany). Effect size estimates were derived from BV/TV data reported in our previous study (Ju et al., 2020), in which exercise interventions produced large differences compared with control conditions (Cohen's d > 2.8). Assuming an alpha level of 0.05 and a statistical power of 80%, the estimated minimum sample size was seven animals per group. Therefore, the group size of 10 animals adopted in the present study was considered sufficient to detect exercise‐induced changes in trabecular bone.

### 2.6 Statistical analysis

Data were analyzed using IBM SPSS Statistics for Windows, version 26 (SPSS Inc., Chicago, IL). Data are presented as mean ± SD. Normality and homogeneity of variances were assessed using Shapiro–Wilk and Levene's tests; logarithmic transformation was applied if assumptions were violated. Only predefined technical failures were excluded, and missing data were analyzed as complete cases. Two‐way ANOVA with exercise (CON, RUN, JUM) and detraining (0, 12, 24 weeks) as factors was performed, followed by Tukey's post hoc test. Significant exercise × detraining interactions were further examined with simple main effects. Pairwise effect sizes were calculated using Cohen's d and interpreted as small (0.2), moderate (0.5), or large (0.8). Mean differences (MD) are reported as absolute values with 95% confidence intervals (CI), and statistical significance was set at *p* < 0.05.

## 3 RESULTS

### 3.1 General characteristics of rats

The general characteristics of the rats are shown in Table 1. All groups exhibited an increase in body weight following the exercise period and during each detraining phase; however, no significant differences were observed between the sedentary control and either exercise group. Calf muscle weight was measured to assess the effects of running and jump exercise on skeletal muscle. However, no significant differences were found among the sedentary control and the two exercise groups at the end of the experiment. Femoral length was also comparable across groups. Thus, running and jump exercise did not affect either skeletal muscle weight or femoral length.

**TABLE 1:** Body weight, calf muscles weight, and femoral length in experimental rats.

	0‐week detraining			12‐weeks detraining			24‐weeks detraining		
	CON	RUN	JUM	3CON	R3DT	J3DT	6CON	R6DT	J6DT
Initial Body weight (g)	310.3 ± 09.48	305.0 ± 09.25	306.2 ± 12.37	303.8 ± 13.30	304.3 ± 05.86	308.3 ± 11.58	310.1 ± 08.89	303.5 ± 09.82	300.7 ± 08.33
Final Body weight (g)	401.5 ± 13.16	394.9 ± 26.49	403.7 ± 13.55	457.1 ± 41.91**	474.7 ± 22.68###	474.1 ± 22.87†††	496.6 ± 35.69***	508.3 ± 40.58###	501.9 ± 43.33†††
Calf muscles weight (g)	2.03 ± 0.16	1.98 ± 0.19	1.96 ± 0.14	2.20 ± 0.20	2.30 ± 0.08##	2.39 ± 0.29†††	2.47 ± 0.19***	2.45 ± 0.18###	2.40 ± 0.20†††
Femoral length (mm)	37.00 ± 1.50	36.47 ± 1.41	36.77 ± 1.10	39.00 ± 0.68***	38.92 ± 0.53###	39.22 ± 0.51†††	39.58 ± 0.69***	39.74 ± 1.17###	39.84 ± 0.64†††

*Note*: All values are presented as mean ± SD. *n* = 10 per group. Statistical significance was determined by two‐way ANOVA with Tukey's post hoc test. Groups: RUN, running exercise for 8 weeks; JUM, jump exercise for 8 weeks; CON, sedentary control for 8 weeks; R3DT, running exercise followed by 12 weeks of detraining; J3DT, jump exercise followed by 12 weeks of detraining; 3CON, sedentary control for 12 weeks; R6DT, running exercise followed by 24 weeks of detraining; J6DT, jump exercise followed by 24 weeks of detraining; and 6CON, sedentary control for 24 weeks. Significant difference versus CON: **p < 0.01; ***p < 0.001. Significant difference versus RUN: ^##^ *p* < 0.01; ^###^ *p* < 0.001. Significant difference versus JUM: ^†††^ *p* < 0.001.

### 3.2 Effects of exercise on trabecular bone

**FIGURE 2:**
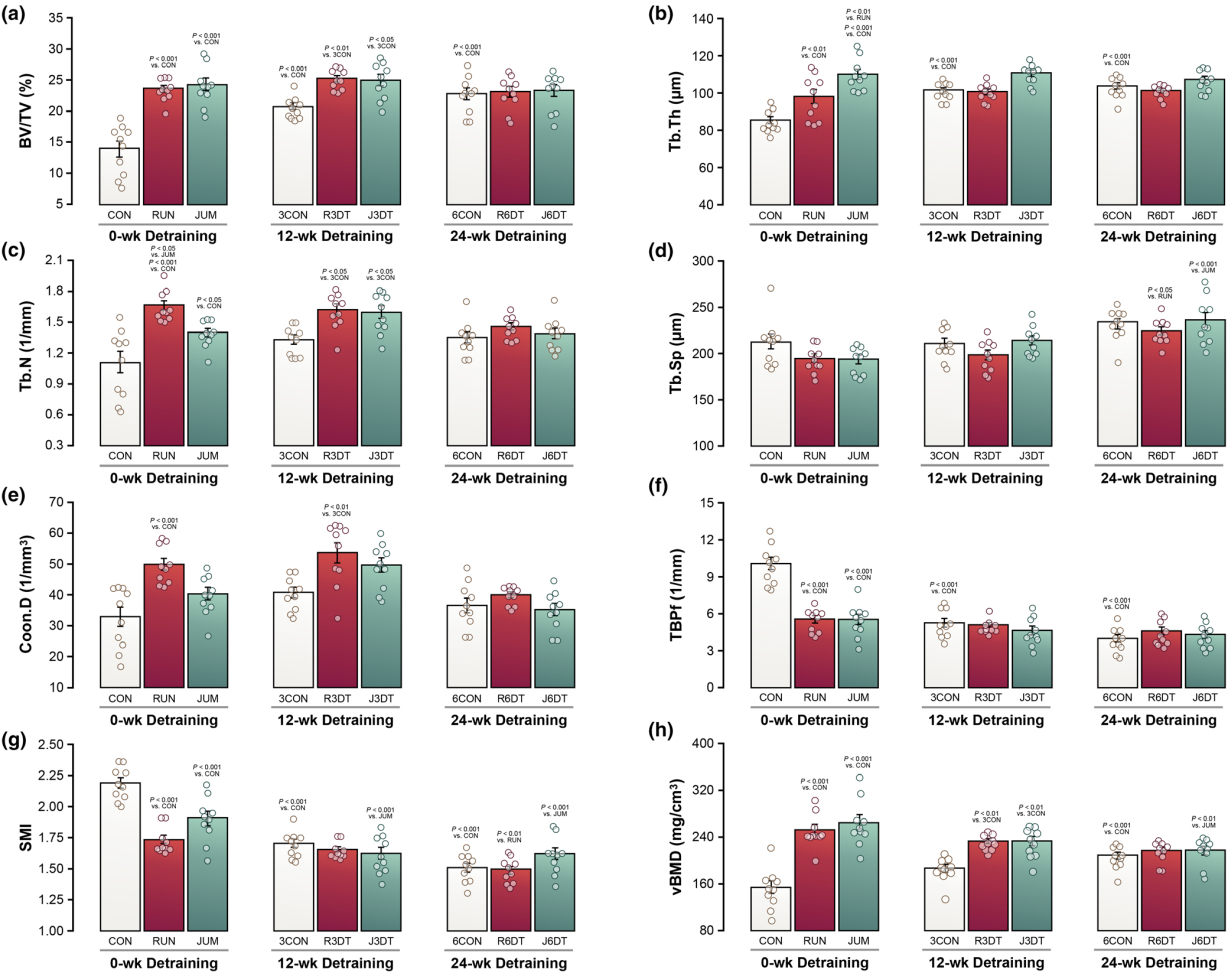
Exercise‐induced changes and detraining‐related alterations in trabecular bone microarchitecture. Groups: RUN, running exercise for 8 weeks (*n* = 10); JUM, jump exercise for 8 weeks (*n* = 10); CON, sedentary control for 8 weeks (*n* = 10); R3DT, running exercise followed by 12 weeks of detraining (*n* = 10); J3DT, jump exercise followed by 12 weeks of detraining (*n* = 10); 3CON, sedentary control for 12 weeks (*n* = 10); R6DT, running exercise followed by 24 weeks of detraining (*n* = 10); J6DT, jump exercise followed by 24 weeks of detraining (*n* = 10); and 6CON, sedentary control for 24 weeks (*n* = 10). Trabecular parameters are shown as: BV/TV, trabecular bone volume fraction (a); Tb.Th, trabecular thickness (b); Tb.N, trabecular number (c); Tb.Sp, trabecular separation (d); Conn.D, connectivity density (e); TBPf, trabecular bone pattern factor (f); SMI, structure model index (g); and vBMD, volumetric bone mineral density (h). All values are presented as mean ± SD. Statistical significance was determined by two‐way ANOVA with Tukey's post hoc test.

**FIGURE 3:**
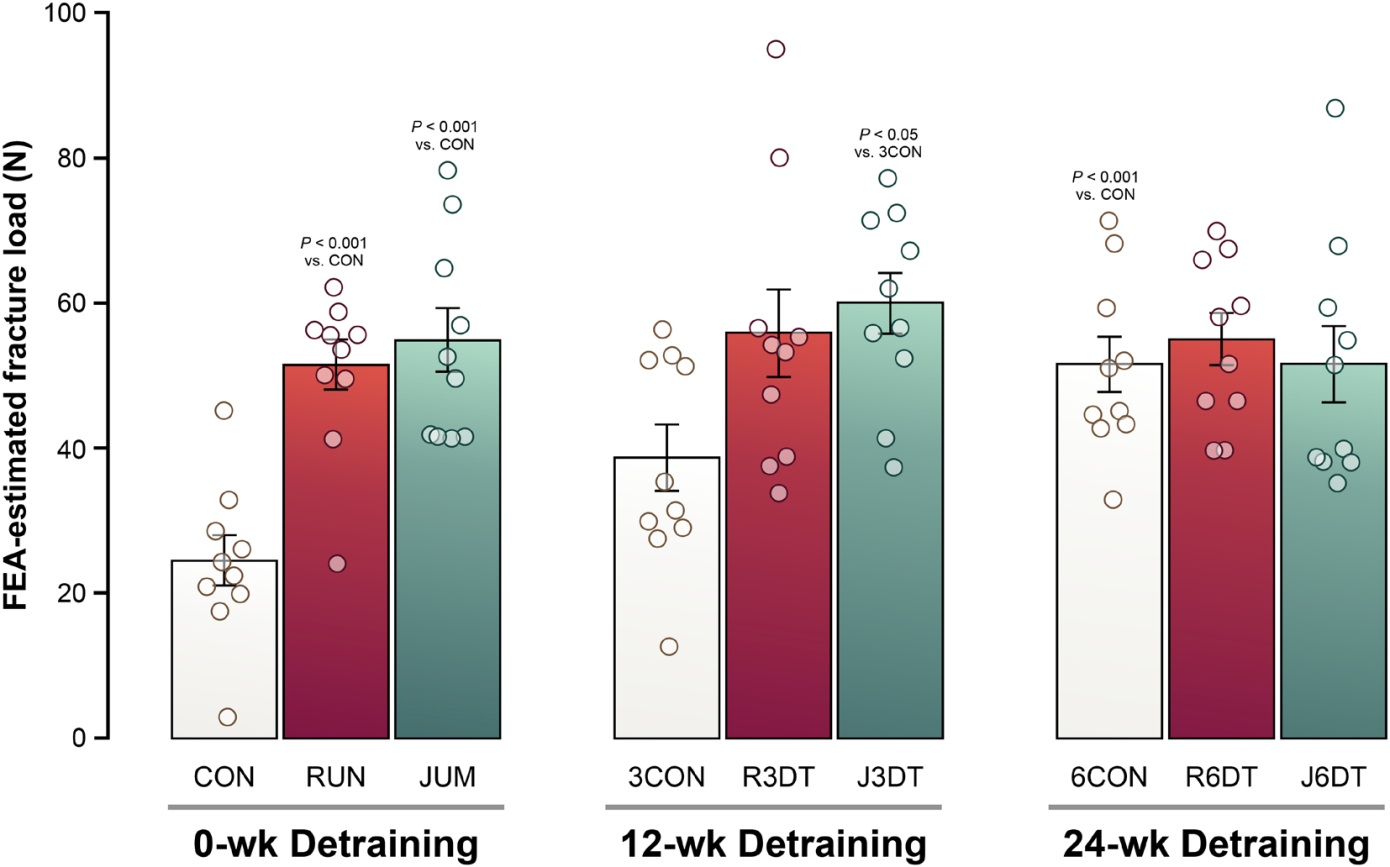
FEA–predicted fracture load following exercise and detraining. Values were calculated based on micro‐CT‐derived trabecular microarchitecture of the femora. Groups: RUN, running exercise for 8 weeks (*n* = 10); JUM, jump exercise for 8 weeks (*n* = 10); CON, sedentary control for 8 weeks (*n* = 10); R3DT, running exercise followed by 12 weeks of detraining (*n* = 10); J3DT, jump exercise followed by 12 weeks of detraining (*n* = 10); 3CON, sedentary control for 12 weeks (*n* = 10); R6DT, running exercise followed by 24 weeks of detraining (*n* = 10); J6DT, jump exercise followed by 24 weeks of detraining (*n* = 10); and 6CON, sedentary control for 24 weeks (*n* = 10). All values are presented as mean ± SD. Statistical significance was determined by two‐way ANOVA with Tukey's post hoc test.

Results for trabecular bone microarchitecture and FEA‐predicted fracture load in the distal metaphysis of the femur are shown in Figures 2 and 3. MD with 95% CI and Cohen's *d* are presented in Table 2 to quantify the magnitude of group differences. At the end of the training period, both RUN and JUM induced marked increases in trabecular bone mass and structural integrity compared with the CON. Specifically, RUN exhibited significantly higher BV/TV, Tb.Th, Tb.N, Conn.D, and vBMD (69%, *p* < 0.001; 15%, *p* < 0.01; 51%, *p* < 0.001; 52%, *p* < 0.001, 64%, *p* < 0.001, respectively), whereas TBPf and SMI (−45%, *p* < 0.001; −21%, *p* < 0.001, respectively) were significantly reduced. JUM produced greater improvements in BV/TV, Tb.Th, Tb.N, and vBMD (73%, *p* < 0.001; 29%, *p* < 0.001; 27%, *p* < 0.05; 72%, *p* < 0.001, respectively), with concomitant reductions in TBPf and SMI (−45%, *p* < 0.001; −13%, *p* < 0.001, respectively). Direct comparison between exercise modalities revealed greater Tb.Th (12%, *p* < 0.01) in JUM, whereas Tb.N (19%, *p* < 0.05) was higher in RUN, indicating modality‐specific adaptations in trabecular microarchitecture. FEA‐predicted fracture load was significantly higher in both RUN (38%, *p* < 0.001) and JUM (27%, *p* < 0.001) compared with CON.

**TABLE 2:** Effects of exercise and detraining on trabecular bone microarchitecture, volumetric bone mineral density, and FEA‐predicted fracture load.

Parameter	Comparison	MD	95% CI	*p* value	Cohen's d
BV/TV	RUN versus CON	−9.66	−13.61 to −5.71	0.001	−3.69
	JUM versus CON	−10.24	−14.18 to −6.29	0.001	−3.49
	J3DT versus 3CON	−4.32	−8.28 to −0.37	0.05	−1.56
	R3DT versus 3CON	−4.63	−8.58 to −0.68	0.01	−1.67
Tb.Th	RUN versus CON	−12.58	−22.29 to −2.87	0.01	−1.85
	JUM versus CON	−24.62	−34.33 to −14.90	0.001	−3.61
	JUM versus RUN	12.04	2.32 to 21.75	0.01	1.77
Tb.N	RUN versus CON	−0.56	−0.82 to −0.30	0.001	−3.07
	JUM versus CON	−0.30	−0.56 to −0.04	0.05	−1.62
	RUN versus JUM	−0.27	−0.53 to −0.01	0.05	−1.45
	J3DT versus 3CON	−0.27	−0.53 to −0.01	0.05	−1.46
	R3DT versus 3CON	−0.29	−0.55 to −0.03	0.05	−1.58
Tb.Sp	R6DT versus RUN	30.14	4.06 to 56.23	0.05	1.65
	J6DT versus JUM	42.43	16.34 to 68.51	0.001	2.32
Conn.D	RUN versus CON	−17.02	−27.28 to −6.75	0.001	−2.36
	R3DT versus 3CON	−12.99	−23.21 to −2.72	0.01	−1.80
TBPf	C3DT versus CON	−16.07	−25.79 to −6.36	0.001	−2.35
	C6DT versus CON	−16.07	−25.79 to −6.36	0.001	−2.35
SMI	J3DT versus JUM	0.29	0.10 to 0.47	0.001	2.24
	J6DT versus JUM	0.29	0.11 to 0.47	0.001	1.86
	R6DT versus RUN	0.24	0.06 to 0.42	0.01	1.86
vBMD	RUN versus CON	−99.14	−136.28 to −62.00	0.001	−3.81
	JUM versus CON	−111.48	−148.62 to −74.34	0.001	−4.28
	J3DT versus 3CON	−46.20	−83.34 to −9.06	0.01	−1.77
	R3DT versus 3CON	−46.23	−83.37 to −9.09	0.01	−1.77
	J6DT versus JUM	47.08	9.94 to 84.22	0.01	1.81
FEA‐predicted fracture load	RUN versus CON	−26.95	−46.75 to −7.16	0.001	−1.94
	JUM versus CON	−30.33	−50.13 to −10.54	0.001	−2.18
	J3DT versus 3CON	−21.39	−41.19 to −1.59	0.01	−1.54

*Note*: Values are mean differences (MD) between groups with corresponding 95% confidence intervals (CI). Negative values indicate higher values in the exercise or detraining group relative to the reference group as specified. Effect sizes were calculated using Cohen's *d* and interpreted as small (0.2), moderate (0.5), or large (0.8). Statistical significance was set at *p* < 0.05. Groups: RUN, running exercise for 8 weeks (*n* = 10); JUM, jump exercise for 8 weeks (*n* = 10); CON, sedentary control for 8 weeks (*n* = 10); R3DT, running exercise followed by 12 weeks of detraining (*n* = 10); J3DT, jump exercise followed by 12 weeks of detraining (*n* = 10); 3CON, sedentary control for 12 weeks (*n* = 10); R6DT, running exercise followed by 24 weeks of detraining (*n* = 10); J6DT, jump exercise followed by 24 weeks of detraining (*n* = 10); and 6CON, sedentary control for 24 weeks (*n* = 10).

Abbreviations: BV/TV, trabecular bone volume fraction; Conn.D, connectivity density; FEA, finite element analysis; SMI, structure model index; Tb.N, trabecular number; Tb.Sp, trabecular separation; Tb.Th, trabecular thickness; TBPf, trabecular bone pattern factor; vBMD, volumetric bone mineral density.

### 3.3 Effects of detraining on trabecular bone

After 12 weeks of detraining, several exercise‐induced trabecular adaptations were preserved. J3DT group showed significantly greater BV/TV, Tb.N, and vBMD than 3CON (21%, *p* < 0.05; 9%, *p* < 0.05; 25%, *p* < 0.01, respectively), whereas Tb.Th showed nonsignificant trends toward higher values. R3DT also showed significantly higher BV/TV, Tb.N, Conn.D, and vBMD when compared with the 3CON (22%, *p* < 0.05; 22%, *p* < 0.01; 32%, *p* < 0.01; 25%, *p* < 0.01, respectively). FEA‐predicted fracture load remained significantly elevated in J3DT (55%, *p* < 0.01) and showed a similar, though nonsignificant, trend in R3DT (45%, *p* < 0.13).

In contrast, after 24 weeks of detraining, trabecular bone parameters in both J6DT and R6DT declined to levels comparable with those of time‐matched sedentary controls, indicating attenuation of exercise‐induced benefits with prolonged cessation of mechanical loading.

### 3.4 Time‐dependent changes in trabecular bone

Compared with the CON, both C3DT (47%, *p* < 0.001; 19%, *p* < 0.001, respectively) and C6DT (63%, *p* < 0.001; 21%, *p* < 0.001, respectively) groups showed significantly higher BV/TV and Tb.Th. Significant increases in vBMD and FEA‐predicted fracture load were observed only in C6DT (36%, *p* < 0.001; 111%, *p* < 0.001, respectively). SMI and TBPf were significantly lower in both C3DT (−22%, *p* < 0.001; −49%, *p* < 0.001, respectively) and C6DT (−55%, *p* < 0.001; −60%, *p* < 0.001, respectively) compared with CON. Tb.Sp was significantly increased in R6DT (16%, *p* < 0.05) and J6DT (22%, *p* < 0.001). Within the exercise groups, SMI was significantly lower in J3DT (−15%, *p* < 0.001) and J6DT (−15%, *p* < 0.001) compared with JUM, whereas in RUN, a significant reduction in SMI was observed only in R6DT (−13%, *p* < 0.01). vBMD was significantly lower in J6DT (−22%, *p* < 0.01) than in JUM.

## 4 DISCUSSION

The primary aim of this study was to empirically examine the hypothesis that jump exercise is more effective than running exercise in maintaining trabecular bone mass following exercise cessation. However, our data did not support the hypothesis that jump exercise would preserve cancellous bone mass in the distal femoral metaphysis for a longer period than running exercise. The increases in trabecular bone mass and strength induced by both running and jump exercise were largely maintained for up to 12 weeks after cessation. With prolonged exercise discontinuation, these benefits gradually aligned with those of sedentary controls, mainly reflecting natural growth and skeletal maturation in the control animals.

Appropriate mechanical loading, such as various types of exercise, can modify trabecular bone structure in rats. In a histomorphometric study, Yeh et al. (1993) reported that trabecular number in the tibial metaphysis of rats after 9 and 16 weeks of running exercise was higher than in controls, whereas trabecular thickness remained unchanged. Using micro‐CT, we also found that moderate treadmill running for 10 weeks increased trabecular bone mass mainly through increases in trabecular number (22%), with only a slight increase in trabecular thickness (8%) in the distal femoral metaphysis of rats (Joo et al., 2003). In contrast, Notomi, Lee, et al. (2000) reported that trabecular thickness in the proximal tibial metaphysis of growing rats increased significantly after 4 and 8 weeks of jump exercise, whereas trabecular number remained essentially unchanged. In another study, they also found that 8 weeks of jump exercise increased trabecular thickness in the lumbar vertebra without notable changes in trabecular number (Notomi, Okazaki, et al., 2000). Interestingly, although different mechanical loads have been suggested to induce trabecular bone with distinct structures and mechanical properties, this aspect has received relatively little attention. In a previous study, we reported for the first time that running and jump exercises have differential effects on trabecular bone structure, with jump exercise promoting trabecular thickness and running being associated with higher trabecular number (Ju et al., 2012). A similar trend was observed in the present study. Furthermore, this is consistent with Berman et al. (2019), who reported that tibial loading and treadmill running differentially improve bone mass, with tibial loading increasing trabecular thickness and treadmill running being associated with higher trabecular number. Despite extensive research, the mechanisms by which different mechanical loads lead to variations in trabecular bone structure remain unclear. One possible explanation for these findings is the qualitative difference in mechanical stimuli between running and jump exercises. Jump exercise involves brief, high‐magnitude impact loading that is relatively concentrated along a specific principal stress direction and may preferentially stimulate bone formation on existing trabeculae, leading to trabecular thickening. In contrast, running provides relatively low‐magnitude but sustained, repetitive, and multidirectional loading, which may distribute mechanical stress across the trabecular network and contribute to the maintenance of trabecular structure and higher trabecular number. Thus, distinct loading modalities may differentially shape trabecular microarchitecture through differences in strain magnitude, distribution, and temporal pattern. This is supported by studies suggesting that variations in strain rate and loading frequency can selectively influence trabecular thickening versus changes in trabecular number, even when bone mass gains are similar (Christiansen et al., 2008; Turner, 1998). Nevertheless, FEA‐estimated fracture load of the distal femoral metaphysis did not differ between jump and running exercises. These results suggest that both types of exercise enhance trabecular bone mass and strength through different structural changes, although these differences do not impact overall trabecular strength.

A previous study demonstrated that a strain of approximately 1500 μɛ (≈10 N/kg) is required to induce a bone formation response during tibial loading (De Souza et al., 2005). In our previous work using a similar jump protocol, vertical ground reaction forces in rats reached approximately 6.3 times body weight (≈23.5 N/kg) (Ju et al., 2022), suggesting that substantially higher mechanical stimuli were imposed than those typically observed during treadmill running. By comparison, in an experiment involving 12‐week‐old rats walking along an instrumented walkway, the average peak vertical force was approximately 0.68 times body weight (≈6.63 N/kg) (Dienes et al., 2022), although the loading conditions were not identical to those used in the present study. Although direct strain measurements were not obtained, these force‐based estimates provide indirect evidence that jump exercise likely imposes substantially greater mechanical strain on bone than treadmill running. Such differences in loading characteristics may underlie the distinct alterations in trabecular microstructure observed in the distal femoral metaphysis of rats.

Exercise during skeletal growth can induce beneficial adaptations in bone size and structure; however, it remains unclear whether these adaptations are maintained after exercise cessation. In a clinical study, Vuori et al. (1994) showed that unilateral limb strength training in young women increased BMD in both the trained and untrained limbs, but BMD declined toward baseline after training had stopped. Dalsky et al. (1988) also demonstrated that short‐ and long‐term weight‐bearing exercise led to significant increases above baseline in lumbar bone mineral content (BMC), but BMC reverted to baseline levels after detraining in postmenopausal women. In an animal study, LeBlanc et al. (1983) showed that the increased rate of total body calcium gain produced by voluntary exercise was reversed after cessation in adult rats. Yeh and Aloia (1990) similarly reported that bone mass gained by treadmill exercise was lost during deconditioning in young female rats after the growth spurt period. Collectively, these longitudinal studies suggest that bone mass gained by exercise may be lost after detraining in adults or in subjects after rapid growth.

Several studies have reported that the beneficial effects of prior exercise can persist despite reductions in activity or even after complete cessation. For example, exercise during growth and early adulthood is associated with sustained cortical bone enlargement and reduced fracture risk later in life (Nilsson et al., 2014). In animal models, jump‐induced gains in cortical bone geometry and strength persisted for up to 24 weeks after detraining, despite gradual declines in BMD (Honda et al., 2008; Umemura et al., 2008). Similarly, brief periods of axial loading produced lasting improvements in ulnar cross‐sectional geometry in rats (Warden et al., 2007). These findings suggest that greater bone thickness or structural adaptation may confer an enhanced capacity to maintain bone mass after exercise cessation. In the present study, trabecular bone mass and strength gained from running and jump exercise remained above sedentary control levels for up to 12 weeks after exercise cessation. This finding differs from previous reports describing rapid bone mass loss following short‐term running interventions (Iwamoto et al., 2000; Yeh & Aloia, 1990), which may be attributable to differences in skeletal site assessed (tibia versus femur) or in measurement methodology (DXA versus micro‐CT). Overall, these results indicate that structural adaptations in both cortical and trabecular bone can persist long after exercise cessation, even when BMD returns toward baseline. This emphasizes the importance of considering bone geometry and microarchitecture, in addition to bone mass, when assessing the long‐term skeletal benefits of exercise.

Exercise‐induced improvements in trabecular bone structure and strength, including increases in bone volume, thickness, and FEA‐estimated fracture load, were largely maintained for up to 24 weeks following cessation of training. Although detraining halted further gains in these parameters, no significant declines were observed, indicating that adaptations acquired through prior mechanical loading are relatively resistant to short‐ to intermediate‐term detraining. The apparent convergence between the jumping and running groups and sedentary controls was not attributable to bone loss in the exercised groups. Rather, it primarily reflected ongoing skeletal growth and maturation in the control animals, which led to measurable increases in trabecular bone parameters over the same period. Therefore, the observed reduction in group differences appears to result mainly from continued development in the controls, rather than from a loss of exercise‐induced skeletal benefits in the jumping and running groups. Taken together, these findings suggest that mechanical loading during periods of rapid growth establishes robust and durable improvements in trabecular bone structure and strength. However, because further bone accrual in the exercised groups plateaued following cessation of loading, continued mechanical stimulation may be required to maintain or enhance skeletal advantages beyond those conferred by normal growth and maturation.

In the present study, 24 weeks after exercise cessation, trabecular thickness in the jump exercise group aligned with that of the running and sedentary control groups. Thus, our results did not support the notion that thicker trabecular bone preserves distal femoral metaphyseal bone mass longer than thinner trabeculae of the same bone mass in rats. Despite a comparable 24‐week exercise cessation period, our findings differed from those of previous studies (Honda et al., 2008; Umemura et al., 2008), which may reflect differences between trabecular and cortical bone morphology. Trabecular bone is generally considered to have a higher turnover rate than cortical bone due to its greater surface area (Lane et al., 1996), suggesting that trabecular bone responds more readily to changes in mechanical stimuli and returns to baseline more quickly after exercise cessation. Because the primary objective of the present study was to examine the persistence of exercise‐induced skeletal adaptations after detraining, subsequent analyses focused primarily on trabecular bone, where clear exercise‐induced structural responses were observed. Cortical bone parameters at the femoral mid‐diaphysis were also evaluated at the end of the training period; however, neither running nor jump exercise produced detectable cortical adaptations at this skeletal site (data not shown). These findings further suggest site‐specific differences in skeletal adaptation to mechanical loading. Supporting this notion, Wallace et al. (2007) demonstrated that running exercise in growing male mice markedly increased periosteal and endocortical perimeters, cortical area, medial‐lateral width, and bending moment of inertia at the tibial diaphysis, whereas the femoral diaphysis showed only minimal changes. These findings indicate that the cortical bone response to mechanical loading is strongly site dependent.

Several methodological limitations of the present study should be considered when interpreting the results. First, mechanical strain and ground reaction forces were not measured during running or jump exercise, leaving potential differences in mechanical loading between groups speculative. Consequently, it cannot be conclusively determined whether variations in strain or impact during exercise underlie the observed differences in distal femoral metaphyseal trabecular microstructure. Second, exercise intensity for both running and jump protocols was not assessed. Achieving comparable loading conditions between running and jump exercises is inherently difficult. In the present study, exercise protocols were based on our previous work to induce comparable increases in trabecular bone mass and strength from both exercise modalities (Ju et al., 2012). Consistent with our expectations, although running and jump exercises differentially affected trabecular microarchitecture, trabecular bone volume fraction, vBMD, and FEA‐derived bone strength were comparable between the two modalities. Finally, DXA was not performed in this study. Micro‐CT provides high‐resolution analysis of trabecular bone, focusing on specific regions, whereas DXA measures whole‐bone mineral content. The limited scan volume of micro‐CT makes direct comparison with DXA difficult and should be considered when interpreting the results.

The present findings have several practical implications. Exercise during growth induces substantial improvements in trabecular bone mass and strength; however, these benefits are not indefinitely maintained after cessation, underscoring the importance of sustaining regular physical activity during adolescence. Different exercise modalities induce distinct patterns of trabecular adaptation, with jump exercise preferentially increasing trabecular thickness, whereas running is associated with higher trabecular number. These findings suggest that incorporating a variety of mechanical loading patterns, including impact‐type activities, may optimize skeletal development. Collectively, our results support the promotion of continued weight‐bearing and impact‐based exercise in children and adolescents to enhance long‐term bone health and reduce future osteoporosis risk.

## 5 CONCLUSION

In conclusion, we demonstrated that in the distal femoral metaphysis of rats, jump exercise primarily increased trabecular thickness, whereas running exercise was associated with higher trabecular number. Both exercise modalities significantly improved trabecular bone volume fraction and strength, and these benefits were largely maintained for up to 12 weeks after cessation. Over a prolonged period without exercise, the benefits in both exercise groups were slightly reduced, and the gap between exercised and sedentary control animals largely disappeared, mainly reflecting skeletal growth and maturation in the controls. These findings indicate that exercise‐induced trabecular adaptations, including thickening associated with jump exercise and increases in trabecular number associated with running, are partially attenuated following exercise cessation, with differences in trabecular bone mass relative to age‐matched controls diminishing over time.

## AUTHOR CONTRIBUTIONS

**Yong‐In Ju:** Conceptualization; formal analysis; investigation; methodology; visualization. **Teruki Sone:** Conceptualization; methodology; supervision.

## FUNDING INFORMATION

No funding information provided.

## CONFLICT OF INTEREST STATEMENT

The authors declare no conflict of interest.

## ETHICS STATEMENT

All experimental procedures were approved by the Laboratory Animal Care and Use Committee of Kawasaki University of Medical Welfare (Approval No. 10‐011).

## Data Availability

The authors confirm that all data underlying the findings are fully available without restriction. All relevant data are included within the article.
